# Supplementary material for: A fraction of Pueraria tuberosa extract, rich in antioxidant compounds, alleviates ovariectomized-induced osteoporosis in rats and inhibits growth of breast and ovarian cancer cells
Source: PLoS One. 2021 Jan 14;16(1):e0240068. doi: 10.1371/journal.pone.0240068 (PMC7808586; doi:10.1371/journal.pone.0240068)
Supplement: S1 Table — (DOC) [file pone.0240068.s001.doc]

**S1 Table. Chemical compounds in FRAC from *Pueraria tuberosa* by GC/MS analysis. Results of the GC/MS analysis of the fraction rich in antioxidant compounds (S1 Table) are available as supporting information.**

| **Serial No.** | **Peak RT (min)** | **Peak area** | **Peak area %** | **Peak height** | **Compound identified** | **Molecular formula** | **Molecular weight** |
| --- | --- | --- | --- | --- | --- | --- | --- |
|  | 4.39 | 35587858.15 | 1.90 | 6866710.47 | Hexadecanoic acid, 14-hydroxy-16, 16-dimethoxy-14-methyl-,  ethyl ester | C_21_H_42_O_5_ | 375 |
|  | 4.57  24.06 24.54 26.08 26.29 | 75859493.04  39850802.46  15735092.18  64847866.49  48279055.77 | 4.04  2.12  0.84  3.46  2.57 | 4582623.37  3031031.37  2448241.95  4616697.31  4939056.17 | 9,12,15-octadecatrienoic acid, 2,3-bis [(trimethylsilyl)oxy]propyl ester, (Z,Z,Z)- | C_27_H_52_O_4_Si_2_ | 497 |
|  | 5.29 | 108332678.92 | 5.78 | 7023152.90 | 4,25-secoobscurinervan-4-one,  O-acetyl-22-ethyl-15,16-dimethoxy-,(22à) | C_27_H_36_N_2_O_6_ | 485 |
|  | 7.25 | 28233544.67 | 1.51 | 3339516.04 | Cyclotetrasiloxane, octamethyl- | C_8_H_24_O_4_Si_4_ | 297 |
|  | 9.85 | 29493008.92 | 1.57 | 9302963.70 | Cyclopentasiloxane, decamethy- | C_10_H_30_O_5_Si_5_ | 371 |
|  | 11.43 | 96708603.95 | 5.16 | 12139665.74 | 5-(hydroxymethyl)-2-  (dimethoxymethyl)furan | C_8_H_12_O_4_ | 172 |
|  | 12.39 | 8863528.45 | 0.47 | 3821870.49 | Cyclohexasiloxane, dodecamethyl- | C_12_H_36_O_6_Si_6_ | 445 |
|  | 14.66 | 10560373.07 | 0.56 | 3539807.59 | Cycloheptasiloxane, tetradecamethyl- | C_14_H_42_O_7_Si_7_ | 519 |
|  | 16.69 | 8111960.29 | 0.43 | 3450046.48 | Cyclooctasiloxane, hexadecamethyl- | C_16_H_48_O_8_Si_8_ | 593 |
|  | 18.26 | 14224351.42 | 0.76 | 4575637.14 | 3-hydroxybutyric acid,  2-(2-t-butoxycarbonyl-1-methyl-ethoxycarbonyl)-1-methyl-ethyl ester | C_16_H_28_O_7_ | 332 |
|  | 18.43 | 7097007.94 | 0.38 | 2595925.30 | Cyclononasiloxane, octadecamethyl- | C_18_H_54_O_9_Si_9_ | 667 |
|  | 19.02 | 9289498.16 | 0.50 | 2297153.81 | Hexadecanoic acid, methyl ester | C_17_H_34_O_2_ | 270 |
|  | 19.19 | 32081653.04 | 1.71 | 13390506.18 | Dibutyl phthalate | C_16_H_22_O_4_ | 278 |
|  | 20.69 | 10815885.27 | 0.58 | 3600405.35 | Trans-13-octadecenoic  acid, methyl ester | C_19_H_36_O_2_ | 296 |
|  | 20.93 25.43 | 148369520.85  54084810.73 | 7.91  2.88 | 10179949.55  6470001.25 | Tetratriacontane | C_34_H_70_ | 479 |
|  | 22.83 | 120415631.83 | 6.42 | 9333683.54 | Stigmasterol | C_29_H_48_O | 413 |
|  | 23.30, 24.90 | 101698680.25  447687254.73 | 5.42  23.87 | 7813646.11  42398394.49 | β-Sitosterol | C_29_H_50_O | 415 |
|  | 24.37 | 38187645.46 | 2.04 | 11498465.41 | Phthalic acid, di(2-propylpentyl) ester | C_24_H_38_O_4_ | 391 |
|  | 25.77 | 33962886.51 | 1.81 | 2690633.26 | 1-monolinoleoylglycerol  trimethylsilyl ether | C_27_H_54_O_4_Si_2_ | 499 |
|  | 26.58 | 131301047.05 | 7.00 | 25532493.79 | 13-docosenamide, (Z)- | C_22_H_43_NO | 338 |
|  | 27.88 | 31980675.83 | 1.71 | 2451448.98 | Stigmasta-3,5-dien-7-one | C_29_H_46_O | 411 |
|  | 29.45 | 78962415.54 | 4.21 | 4528908.53 | Octadecane, 3-ethyl-5-(2- ethylbutyl)- | C_26_H_54_ | 367 |
|  | 31.20 | 45014291.98 | 2.40 | 2348721.40 | 5H-cyclopropa[3,4]benz[1,2-e]  azulen-5-one,  2,4a,9,9a-tetrakis(acetyloxy)-3,[(acetyloxy) methyl]1,1a,  1b,2,3,4,4a,7a,7b,8,9,9a-dodecahydro-2,7b-dihydroxy-1,1,6,8-tetramethyl-,  [1aR-(1aà,1bá,2à,3á,4aá,7aà,7bà,8à,9á,9aà)]- | C_30_H_40_O_13_ | 609 |
