# Supplementary material for: A fraction of Pueraria tuberosa extract, rich in antioxidant compounds, alleviates ovariectomized-induced osteoporosis in rats and inhibits growth of breast and ovarian cancer cells
Source: PLoS One. 2021 Jan 14;16(1):e0240068. doi: 10.1371/journal.pone.0240068 (PMC7808586; doi:10.1371/journal.pone.0240068)
Supplement: Attachment — Submitted filename: Responses to reviewers.doc [file pone.0240068.s003.doc]

To

The Editor-in-Chief

PLOS ONE

Subject: Submission of revised manuscript: **Antioxidant enriched fraction from *Pueraria tuberosa* alleviates ovariectomized-induced osteoporosis in rats, and inhibits growth of breast and ovarian cancer cell lines *in vitro***

Dear Editor-in-Chief,

Thank you for sending the reviewer’s comments regarding our submitted manuscript titled “**Antioxidant enriched fraction from *Pueraria tuberosa* alleviates ovariectomized-induced osteoporosis in rats, and inhibits growth of breast and ovarian cancer cell lines *in vitro***”. We would like to thank the editor, and reviewers for their constructive comments.

We have carefully considered all the minor/major comments made by the editor/reviewer, and extensively revised the manuscript accordingly. The reviewers suggested for some more experimental work, which is extremely difficult as the animal experiments have been carried out in 2016. We think that there is great amount of data presented in the manuscript to support the conclusion from the study. Therefore, we request you to kindly consider the revised manuscript to be published in the journal.

Below we present our point by point responses to the reviewer/editorial comments:

| **Reviewer/Editorial Comments** | **Response** |
| --- | --- |
| "Main title: "Antioxidant enriched fraction" has no meaning. Instead "Antioxidant" it can stand "Antioxidant compounds". Moreover,  "enriched" sounds like the authors somehow added antioxidant compounds to the fraction they have investigated. Instead of "enriched", "rich"(if they are clearly reach) or "containing" should read (throughout the text). And finally, the authors did not investigate a fraction of P. tuberosa, but a fraction of its extract." | We have corrected "Antioxidant enriched fraction" to "Antioxidant-rich fraction" throughout the manuscript.  The title is changed as: **Antioxidant-rich fraction from *Pueraria tuberosa* alleviates ovariectomized-induced osteoporosis in rats, and inhibits growth of breast and ovarian cancer cells** |
| 1) Please ensure that your manuscript meets PLOS ONE's style requirements, including those for file naming. The PLOS ONE style templates can be found at | Corrected as per the PLOS ONE style templates. |
| 2) PLOS requires an ORCID iD for the corresponding author in Editorial Manager on papers submitted after December 6th, 2016. Please ensure that you have an ORCID iD and that it is validated in Editorial Manager. To do this, go to ‘Update my Information’ (in the upper left-hand corner of the main menu), and click on the Fetch/Validate link next to the ORCID field. This will take you to the ORCID site and allow you to create a new iD or authenticate a pre-existing iD in Editorial Manager. | ORCID iD information is added. |
| 3) Please include a copy of Table 3 which you refer to in your text on page 24. | Table 3 was mentioned in the MS by mistake and now it has been corrected as the supplementary material S1. |
| 4) Please include captions for your Supporting Information files at the end of your manuscript, and update any in-text citations to match accordingly. | Captions are included and updated the in-text citations |
| 5) We noticed there is a bit of text overlap with this previously published paper of yours: [https://doi.org/10.1016/j.sajb.2018.05.033,](https://www.rediffmail.com/cgi-bin/red.cgi?red=https%3A%2F%2Fdoi.org%2F10.1016%2Fj.sajb.2018.05.033%2C&isImage=0&BlockImage=0&rediffng=0&rdf=BSMCdwVjBWhTb1I0A2I%3D&rogue=f23f6ab8cdb5b3787ca2ae48f0f0fffbed6cc90a&els=c4bd02aa6c83b91b8326be727f25d915) which will need to be addressed. In your revision ensure you cite all your sources (including your own work), and quote or rephrase any duplicated text outside the methods section. Further consideration is dependent on these concerns being addressed. | We have extensively edited, rephrased the revised manuscript for any text overlap with the previous paper. |
| Reviewer's Responses to Questions |  |
| 1. Is the manuscript technically sound, and do the data support the conclusions? The manuscript must describe a technically sound piece of scientific research with data that supports the conclusions. Experiments must have been conducted rigorously, with appropriate controls, replication, and sample sizes. The conclusions must be drawn appropriately based on the data presented. | Reviewer #1: Yes  Reviewer #2: Partly  Changes and corrections have been made in the manuscript to address the comment for Reviewer #2 |
| 2. Has the statistical analysis been performed appropriately and rigorously? | Reviewer #1: Yes  Reviewer #2: Yes |
| 3. Have the authors made all data underlying the findings in their manuscript fully available? | Reviewer #1: Yes  Reviewer #2: Yes |
| 4. Is the manuscript presented in an intelligible fashion and written in standard English?  PLOS ONE does not copyedit accepted manuscripts, so the language in submitted articles must be clear, correct, and unambiguous. Any typographical or grammatical errors should be corrected at revision, so please note any specific errors here. | Reviewer #1: No  Reviewer #2: No  The manuscript is extensively edited to improve the language. The typographical or grammatical errors have also been corrected. |
| 5. Review Comments to the Author |  |
| Reviewer #1: The manuscript is a good research area. However, there are few grammatical errors that must be check in the manuscript. All comments raised have been uploaded for authors. The basis for the dosages used in the methodology must be provided by authors. | We thank the reviewer for making inspiring comments. We have gone through the manuscript very carefully and corrected grammatical, and spelling mistakes as per the suggestions. The basis for the dose use is provided below. |
| 1) Why and what is the basis for this dose selection? | No mortality or any signs of moribund status were found at this dose (2000 mg/kg). Therefore, the LD_50_ cut-off is 5000 mg/kg (category 5 in the Globally Harmonized Classification System). Usually, 1/5^th^ and 1/10^th^ of the LD_50_ value can be used for animal experimentation and the dose selected (very small dose compared to LD_50_ cut-off, 5000 mg/kg) for screening antiosteoporotic activity, where the treatment was for 90 days. We have not observed and mortality of signs of toxicity during this 90 days period also. This information is now provided in the manuscript (page #, lines #-#) |
| 2) In the molecular docking methodology, it was necessary to detail more, for example, which values of the grid box (x, y and z). | 1x7R: 15.587, 32.224, 22.304  1x76: 29.554, 37.747, 38.951  This information is now provided in the manuscript (page 14, lines 284-285) |
| No proper methodology in ligand preparation. Provide detailed methodology. | 3D conformer (.sdf) of daidzein and genistein were downloaded from PubChem website and used as ligand. FlexXLeadIT 2.1.8 of BiosolveIT uses only 3D conformer of ligands. This information is now provided in the manuscript (page 14, lines 292-293)  Genistein: <https://pubchem.ncbi.nlm.nih.gov/compound/5280961>  Daidzein:  <https://pubchem.ncbi.nlm.nih.gov/compound/5281708>  The information is added in the method section (page#, lines #-#) |
| Why was a reference drug not used to compare with the two compounds used for docking studies? | 5-hydroxy-2-(4-hydroxyphenyl)-1-benzofuran-7-carbonitrile was used as internal ligand in 1X76 (ER-α). Genistein was the internal ligand of 1X7R (ER-β), Figure 10 mentioned in page 22-23. Further, As per earlier literature, estradiol (a reference drug) has docking score of -18 and -17 into estrogen receptor α and β active site, respectively, which is mentioned in the discussion section (Page 27, line 548-549). |
| Also, authors should provide the ADMET profile for these compounds as well as the Drug likeness prediction | The ADMET profile of the two compounds are now provided in the manuscript (materials section page 14-15, and results section page 23-24) |
| Reviewer #2: The present submission claimed that AEF may alleviate bone loss through its anti-oxidant activity. However, I have a few concerns about the submission. |  |
| (1) Docking study demonstrated AEF may bind to ERa and ERb. Therefore, ER expression should be examined in the bone. | The docking study was done to support the data obtained in animal study and we found good interaction of daidzein and genistein with both ER-α and ER-β. However, ER expression could be carried out in future. We have mentioned it in the revised manuscript (page 22, line 449). |
| (2) The uterus weight relative to body weight should be determined. | The weight ratios are: Gr-I (0.1896%); Gr-II (0.0457%); Gr-III (0.1124%); Gr-IV (0.0734%); Gr-V (0.0918%). We have now included this numbers in the revised manuscript (page 20, line 405-407) |
| (3) The authors claimed that AEF may inhibit hyperlipidemia. However, the histological staings in the provided figure appeared that there is less fat disposition in the femur. In addition, oil red O staings should be evaluated in the bone. Moreover, what is the alterations of serum HDL and LDL? | We thank the reviewer for pointing this out. We made the statement based on the ability of AEF to reduce the OVX-induced increased level of triglycerides and total cholesterol. We have not measured the serum HDL and LDL levels at the time of animal experiment. |
| (4) The redox status should be tested in the bone. | Thanks for the suggestion. As we have not measured the balance between oxidants and antioxidants during the experiment. |
| (5) If possible, the BMD and bone microstructure should be evaluated. | We thank the reviewer for the great suggestion. But we could not evaluate the BMD and bone microstructure due to lack of facility at the time of the animal experiment. |
| (6) The P value should be followed when the authors mentioned the result is significant. | We thank the reviewer for the excellent suggestion. We have now provided the information in the manuscript (results section in pages 17-20). |
| (7) Please provide the batch number for the kits and chemicals. | Thanks to the reviewer for the suggestion. We have now provided the information here, but not mentioned in the MS. Currently, we do not have the batch number of some of the reagents/chemicals as they were over and not provided here.  3-(4,5-dimethylthiazol-2-yl)-2,5-diphenyltetrazoliumbromide (MTT): lot #11206BE  Dimethyl sulfoxide (DMSO): Lot # S0154  Phosphate Buffered Saline (PBS): Lot # 11419005  Xylazine: FFK8002  Ketamine: 382053  Gentamicin: AHF0051  Calcium kit: CAO-020  Phosphorus kit: PHO-L-B1002  Urea kit: URE-L-B1010  Alkaline phosphatase kit: ALP-L-B1086  Cholesterol kit: HDL-L-B1046  Triglyceride kit: TGR-L-B1106 |
| (8) Grammatical mistakes have been found in the MS. | We have searched and corrected the grammatical mistakes Throughout the manuscript |
| (9) Please eliminate the description of the anticancer. The current data did not support the assertion. | The Antioxidant-Rich Fraction (ARF) has the potential for treatment of menopausal osteoporosis. In addition, it has anticancer effect in cancer cells. This is advantageous as treatment with estrogens have the side effect including possibility of developing cancer.To make this clear,we have mentioned this in the Introduction section (page 4, paragraph 2), discussion (page 27, lines 554-559), conclusion (page 30, lines 567-568), and abstract (page 2, lines 44) |

Dr. Swaha Satpathy
