# Supplementary material for: A fraction of Pueraria tuberosa extract, rich in antioxidant compounds, alleviates ovariectomized-induced osteoporosis in rats and inhibits growth of breast and ovarian cancer cells
Source: PLoS One. 2021 Jan 14;16(1):e0240068. doi: 10.1371/journal.pone.0240068 (PMC7808586; doi:10.1371/journal.pone.0240068)
Supplement: Attachment — Submitted filename: Responses to Reviewers.doc [file pone.0240068.s004.doc]

To

The Editor-in-Chief

PLOS ONE

Subject: Submission of revised manuscript: **Antioxidant-rich fraction from *Pueraria tuberosa* alleviates ovariectomized-induced osteoporosis in rats, and inhibits growth of breast and ovarian cancer cells**

Dear Editor-in-Chief,

Thank you for sending the reviewer’s comments regarding our submitted manuscript titled “**Antioxidant-rich fraction from *Pueraria tuberosa* alleviates ovariectomized-induced osteoporosis in rats, and inhibits growth of breast and ovarian cancer cells**”. We would like to thank the editor, and reviewers for their constructive comments. We have carefully considered all the comments made by the editor/reviewer, and revised the manuscript accordingly (pink highlighted). The reviewers suggested for some more experimental work, which is extremely difficult as the animal experiments have been carried out in 2016. We think that there is great amount of data presented in the manuscript to support the conclusion from the study. Therefore, we request you to kindly consider the revised manuscript to be published in the journal.

Below we present our point by point responses to the reviewer/editorial comments:

| **Reviewer/Editorial Comments** | **Response** |
| --- | --- |
| "Antioxidant" is an adjective and cannot present an object. Therefore, a subject cannot be rich in an attribute, but in some item (noun). The expression "Antioxidant compounds" sound like a logical choice as suggested in the previous review round.  The phrase "fraction from *Pueraria tuberosa*" is unaccomplished, vague. Can authors define how would they describe a fraction of a plant? Instead of that, a fraction of **a plant extract** would be more appropriate.  Considering above mentioned, I would suggest transforming the main title into: "A fraction of *Pueraria tuberosa* extract, rich in antioxidant compounds, alleviates ovariectomized-induced osteoporosis in rats and inhibits growth of breast and ovarian cancer cells". The same terminology has to be applied throughout the text. | The title is changed as: **A fraction of *Pueraria tuberosa* extract, rich in antioxidant compounds, alleviates ovariectomized-induced osteoporosis in rats and inhibits growth of breast and ovarian cancer cells** |
| L23: No need to introduce the abbreviation of the genus name, i.e. please delete the bracket. Instead of that, the species authority should be introduced as: *Pueraria tuberosa* (Roxb. ex Willd.) DC. here and also in the L90. In L89: all the Latin names must stand in italics as well as elsewhere in the text. | Corrections have been made throughout the manuscript (Page: 2, 3, 5, 14, 16-22, 25, 27 & 28). |
| Compound names are randomly capitalized. Please do not write compound names with capital letters unless they present trademarks. | Changes have been made in the manuscript (page: 5 and supplementary material) |
| ARF (antioxidant-rich fraction) cannot stand from the reasons stated as above. "Fraction rich in antioxidant compounds" and a matching abbreviation can read. | ARF (antioxidant-rich fraction) has been stated as "Fraction rich in antioxidant compounds" FRAC throughout the manuscript (Page: 2, 3, 5-7, 9-11, 13, 14, 16-22, 25-28). |
| Figures 8 and 9 are vertically stretched out. Can you revert them to the original ratio? | The figures are now in the original ratio |
| **Reviewer's Responses to Questions** |  |
| 1. If the authors have adequately addressed your comments raised in a previous round of review and you feel that this manuscript is now acceptable for publication, you may indicate that here to bypass the “Comments to the Author” section, enter your conflict of interest statement in the “Confidential to Editor” section, and submit your "Accept" recommendation. | Reviewer #1: All comments have been addressed  Reviewer #2: (No Response)  Reviewer#2 suggested to report some parameters related to animal experiment. We thank the reviewer and appreciate for the suggestions. We will definitely consider them in our future studies. At this time, it is difficult to conduct the whole animal experiments for only these parameters (redox status in bone and HDL & LDL). We have acknowledged and mentioned them for future studies in the conclusion section. |
| 2. Is the manuscript technically sound, and do the data support the conclusions?  The manuscript must describe a technically sound piece of scientific research with data that supports the conclusions. Experiments must have been conducted rigorously, with appropriate controls, replication, and sample sizes. The conclusions must be drawn appropriately based on the data presented. | Reviewer #1: Yes  Reviewer #2: Yes |
| 3. Has the statistical analysis been performed appropriately and rigorously? | Reviewer #1: Yes  Reviewer #2: Yes |
| 4. Have the authors made all data underlying the findings in their manuscript fully available?  The [PLOS Data policy](https://www.rediffmail.com/cgi-bin/red.cgi?red=http%3A%2F%2Fwww.plosone.org%2Fstatic%2Fpolicies.action%23sharing&isImage=0&BlockImage=0&rediffng=0&rogue=60dd09aa7adc37be105aedd52c5096ce8610e0bd&rdf=ByEJfAZgAm8FOVM1AGE=) requires authors to make all data underlying the findings described in their manuscript fully available without restriction, with rare exception (please refer to the Data Availability Statement in the manuscript PDF file). The data should be provided as part of the manuscript or its supporting information, or deposited to a public repository. For example, in addition to summary statistics, the data points behind means, medians and variance measures should be available. If there are restrictions on publicly sharing data—e.g. participant privacy or use of data from a third party—those must be specified. | Reviewer #1: Yes  Reviewer #2: Yes |
| 5. Is the manuscript presented in an intelligible fashion and written in standard English?  PLOS ONE does not copyedit accepted manuscripts, so the language in submitted articles must be clear, correct, and unambiguous. Any typographical or grammatical errors should be corrected at revision, so please note any specific errors here. | Reviewer #1: Yes  Reviewer #2: Yes |
| **6. Review Comments to the Author** Please use the space provided to explain your answers to the questions above. You may also include additional comments for the author, including concerns about dual publication, research ethics, or publication ethics. (Please upload your review as an attachment if it exceeds 20,000 characters) |  |
| Reviewer #1: All comments have been addressed. Research ethics was followed as ethical approval was obtained for this study. | We thank the reviewer for encouraging comments. |
| Reviewer #2: The authors partly responsed my comments. However, (1) The redox status should be tested in the bone. (2) The author should explain why did not test HDL and LDL. | We thank the reviewer and appreciate for the suggestions. We will definitely consider them in our future studies. At this time, it is difficult to conduct the whole animal experiments for only these parameters (redox status in bone and HDL & LDL). We have acknowledged and mentioned them for future studies in the conclusion section. |

Dr. Swaha Satpathy
